# Effects of peripheral inflammation on the blood-spinal cord barrier

**DOI:** 10.1186/1744-8069-8-44

**Published:** 2012-06-18

**Authors:** Dimitris N Xanthos, Isabella Püngel, Gabriele Wunderbaldinger, Jürgen Sandkühler

**Affiliations:** 1 Department of Neurophysiology, Center for Brain Research, Medical University of Vienna, Spitalgasse 4, 1090, Vienna, Austria

**Keywords:** Blood-spinal cord barrier, Capsaicin, Carrageenan, Spinal cord, Occludin, Lumbar, Immunoglobulin, Female, Inflammation

## Abstract

**Background:**

Changes in the blood-central nervous system barriers occur under pathological conditions including inflammation and contribute to central manifestations of various diseases. After short-lasting peripheral and neurogenic inflammation, the evidence is mixed whether there are consistent blood-spinal cord changes. In the current study, we examine changes in the blood-spinal cord barrier after intraplantar capsaicin and λ-carrageenan using several methods: changes in occludin protein, immunoglobulin G accumulation, and fluorescent dye penetration. We also examine potential sex differences in male and female adult rats.

**Results:**

After peripheral carrageenan inflammation, but not capsaicin inflammation, immunohistochemistry shows occludin protein in lumbar spinal cord to be significantly altered at 72 hours post-injection. In addition, there is also significant immunoglobulin G detected in lumbar and thoracic spinal cord at this timepoint in both male and female rats. However, acute administration of sodium fluorescein or Evans Blue dyes is not detected in the parenchyma at this timepoint.

**Conclusions:**

Our results show that carrageenan inflammation induces changes in tight junction protein and immunoglobulin G accumulation, but these may not be indicative of a blood-spinal cord barrier breakdown. These changes appear transiently after peak nociception and may be indicative of reversible pathology that resolves together with inflammation.

## Background

The central nervous system (CNS) is separated and protected from the peripheral environment and blood by the blood–brain/spinal cord barriers (BBB/BSCB), homeostatic control mechanisms of specialized microvasculature which include astrocyte endfeet, endothelial cells, tight junctions, and adherens [1,2]. Various neuroinflammatory diseases such as multiple sclerosis, meningitis, Alzheimer’s disease, and ischemia can result in “breakdown” of the BBB [3]. Pathological conditions that are shown to disrupt the BSCB include traumatic spinal cord injury [4], CNS degenerative disorder [5], CNS inflammation [6], or peripheral nerve injury [7]. Breakdown of the blood-CNS barriers will result in leakage of bioactive substances, neurotransmitters, and immune cells from the blood that can contribute to disease progression and impact drug treatments.

In recent years, the complexity of blood-CNS barriers has been shown to involve multiple physical barriers and specific transport components which could potentially be variably modulated in pathological conditions [8]. Acute stimuli or pathology such as seizures [9], radiation exposure [10], acute stress [11], drugs [12], or hyperthermia [13], can transiently increase the permeability of the BBB. Early and selective changes prior to disease onset with specific mechanisms such as rapid activation of immune cells, for example, mast cells, have also been identified in various diseases including contusive brain injury [14], ischemic brain injury [15], and multiple sclerosis [16].

Peripheral inflammatory stimuli have been shown to induce “breakdown” of the BSCB in some studies, however other results have been conflicting. In one study, carrageenan inflammation induced increased Evans Blue dye extravasation at 48 hours post-carrageenan [17], although this was not systematically studied or replicated. In other studies, complete Freund’s adjuvant (CFA) or carrageenan inflammation are apparently not reported to induce Evans Blue dye leakage at 24 hours post-administration [18] or at 72 hours post-CFA administration [19]. However, morphine penetration is apparently increased specifically in the spinal cord after CFA or carrageenan at 24 hours post-administration [18]. It has also been shown that electrical nerve stimulation at C-fiber intensity or direct application of capsaicin onto the nerve can cause Evans Blue dye leakage in the entire spinal cord at 24 hours post-stimulation [20]. However, in another study, peripherally-injected formalin induces BSCB changes as evidenced by albumin extravasation beginning at 4–6 days, but peaking at 7–10 days post-administration [21] suggesting that this may not be directly related to nociceptive behaviors or hyperalgesia maintenance. It may be speculated that changes in the BSCB particularly in peripheral inflammation may be related to the particular timepoints, particular mechanisms or sensitivity assessed by the various methods used, and characteristics of the animal model. In addition, there may be sex, or strain differences in BSCB disruption as seen in some studies in the brain [22-24] which may affect results in animal models of inflammatory pain. There has been no study using multiple methods at different timepoints to assess changes in the BSCB after peripheral inflammation.

In the current study, we measured changes in the BSCB after intraplantar capsaicin and carrageenan in both male and female rats using three different approaches: changes in morphology of the tight junction protein occludin, endogenous immunoglobulin (IgG) parenchymal permeation, and small size exogenously administered sodium fluorescein (NaFl) permeation. We used multiple early and late timepoints after capsaicin and carrageenan in the hopes of also determining whether changes may be identified as initiating or maintaining the apparent peripheral inflammation and expected nociception. Contrary to expected, we found transient changes using some methods to assess the blood-spinal cord barrier that occurred at a later timepoint not associated with peak nociception. Specifically, our results show changes in spinal occludin protein morphology and presence of IgG in endothelial cells and nervous tissue at 72 hours post-carrageenan in male and female rats, but no major lumbar spinal cord structural changes or increased penetration of exogenously injected small-molecule fluorescent dye.

## Results

### Effect of capsaicin and carrageenan on nociception and spinal tight junction protein occludin

**Figure 1:**
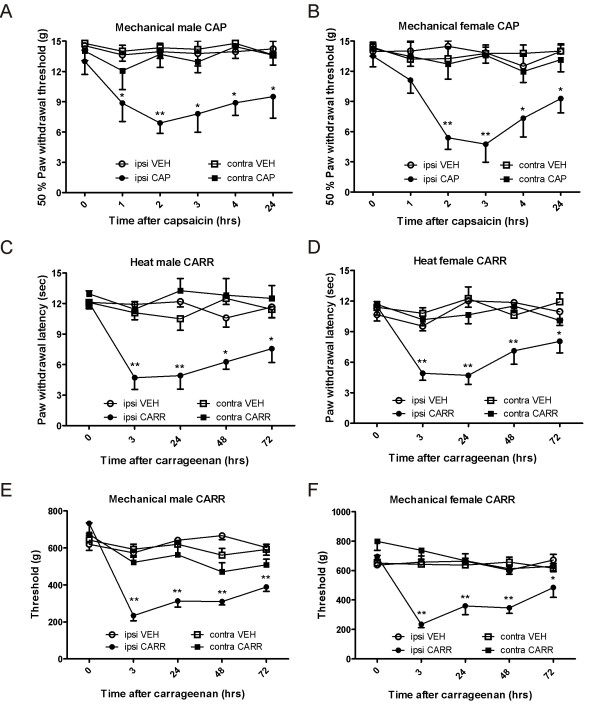
**Intraplantar capsaicin and carrageenan result in significant nociception in both male and female rats.** Intraplantar capsaicin induces significant ipsilateral mechanical hyperalgesia for at least 24 hours post-administration in both male (**A**) and female (**B**) rats, as compared to vehicle injection. Intraplantar carrageenan induces significant ipsilateral heat hyperalgesia and mechanical hyperalgesia in both male (**C**, **E**) and female (**D**, **F**) rats, as compared to vehicle injection. **Abbreviations:** vehicle (VEH), capsaicin (CAP), carrageenan (CARR).

As expected, intraplantar capsaicin induced significant mechanical hyperalgesia as measured with von Frey hairs in the ipsilateral hindpaw in both male and female rats (Figure 1A and Figure 1B), as compared to vehicle injection. Two-way repeated measures analysis of variance (ANOVA) revealed a highly significant effect of group in male (F_3, 100_ = 36.7; P < 0.001) and female rats (F_3, 100_ = 34.8; P < 0.001). Post hoc analysis showed significant differences at all time points tested after capsaicin in the ipsilateral hindpaw as compared to vehicle in male rats (P < 0.05 and P < 0.001 at 2 hours) and at the 2, 3, 4, and 24 hour timepoints after capsaicin in female rats (P > 0.05 and P < 0.001 at 2 and 3 hours). All animals injected with capsaicin also showed significant flinching behaviors for up to 10 minutes post-injection.

Also as expected, intraplantar carrageenan induced significant thermal hyperalgesia (Figure 1C and Figure 1D), as measured by the Hargreaves plantar test and significant mechanical hyperalgesia (Figure 1E and Figure 1F), as measured using the calibrated forceps test in both male and female rats. For thermal testing, two-way repeated measures ANOVA revealed a highly significant effect of group in male (F_3, 80_ = 40.2; P < 0.001) and female rats (F_4, 80_ = 36.2; P < 0.001). Post hoc analysis showed significant differences at all time points tested after carrageenan in the ipsilateral hindpaw as compared to vehicle in male rats (P < 0.05 and P < 0.001 at 3 hours and 24 hours) and female rats (P < 0.05 and P < 0.001 at 3 hours, 24 hours, and 48 hours). For mechanical testing, two-way repeated measures ANOVA also revealed a highly significant effect of group in male (F_3, 80_ = 32.5; P < 0.001) and female rats (F_3, 80_ = 38.7; P < 0.001). Posthoc analysis showed significant differences after carrageenan as compared to vehicle at all time points tested in the ipsilateral hindpaw in male rats (P < 0.05 and P  < 0.001 at 3 hours, 24 hours, 48 hours, and 72 hours) and female rats (P < 0.05 and P < 0.001 at 3 hours, 24 hours, and 48 hours).

**Figure 2:**
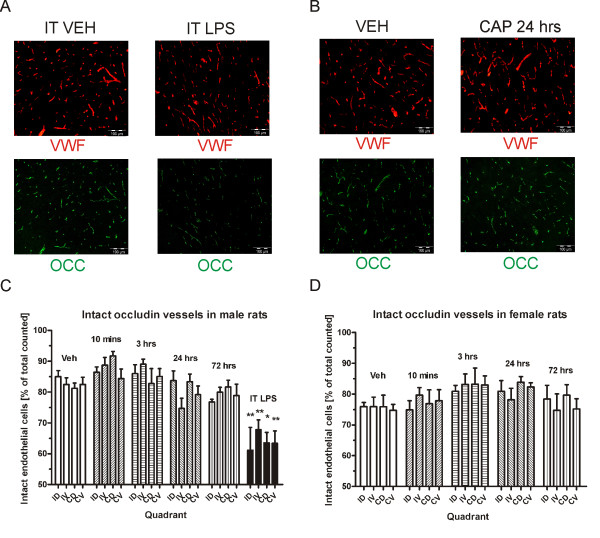
**LPS but not intraplantar capsaicin results in significant changes in spinal tight junction protein occludin.** Intrathecal LPS injection (**A**) but not intraplantar capsaicin (**B**) induces disturbed occludin morphology in lumbar spinal cord. Number of intact vessels are significantly decreased in ipsilateral dorsal, ipsilateral ventral, contralateral dorsal, and contralateral ventral spinal cord quadrants after intrathecal LPS, but not at various timepoints after capsaicin in either male (**C**) or female (**D)** rats. **Abbreviations:** intrathecal (IT), lipopolysaccharide (LPS), von Willebrand Factor (VWF), occludin (OCC) ipsilateral dorsal (ID), ipsilateral ventral (IV), contralateral dorsal (CD), contralateral ventral (CV), others as in Figure 1.

Based on the behavioral timecourse, we chose peak and late timepoints after capsaicin and carrageenan which would allow us to examine the co-occurence of nociception and changes in the BSCB. We systematically examined the morphology of the spinal tight junction protein occludin in male and female rats at various timepoints. Double immunolabeling for occludin protein and the endothelial cell marker von Willebrand factor (VWF) was performed in lumbar spinal cord sections. We then counted the number of intact occludin protein overlapping with VWF staining. As a positive control, intrathecal lipopolysaccharide (LPS) induced clear disturbance of occludin staining with endothelial cells showing lost or diffuse (“foggy”) staining (Figure 2A). We then studied if occludin was altered after 1% capsaicin inflammation at various timepoints. There was no apparent disturbed occludin in endothelial cells after capsaicin treatment at neither 10 minutes, 3 hours, 24 hours, or 72 hours post-injection (Figure 2B). In the four ipsilateral/contralateral and dorsal/ventral quadrants, a total of 8065 vessels were counted in the 6 male vehicle/capsaicin/LPS groups (mean of 1344 ± 98.3 per group) and 5956 vessels in the female vehicle/capsaicin treatment groups (mean of 1191 ± 58.9 per group) in a randomized and blinded fashion. In male rats, two-way ANOVA to analyze the percentage of disturbed occludin protein in endothelial cells revealed a significant effect of group (F_5, 88_ = 59.03; P < 0.001), but not region or interaction (P > 0.05). The posthoc test showed a significant difference between the vehicle group and the intrathecal LPS-treated group in all 4 quadrants: the ipsilateral dorsal, contralateral dorsal and ventral (P < 0.001) and ipsilateral ventral regions (P > 0.05) (Figure 2C). There were no significant differences between vehicle and any of the capsaicin timepoints (P > 0.05). When comparing capsaicin treatments in females, ANOVA revealed a significant effect of group (F_4, 64_ = 15.76; P < 0.05) but no effect or region or interaction (Figure 2D) (P > 0.05). However, post-hoc tests did not reveal any significant differences between groups (P > 0.05). Further individual group analysis by one-way ANOVA also revealed no significant differences between groups within particular regions (P > 0.05). Hence, it can be concluded that intraplantar capsaicin does not induce changes in lumbar spinal occludin protein.

**Figure 3:**
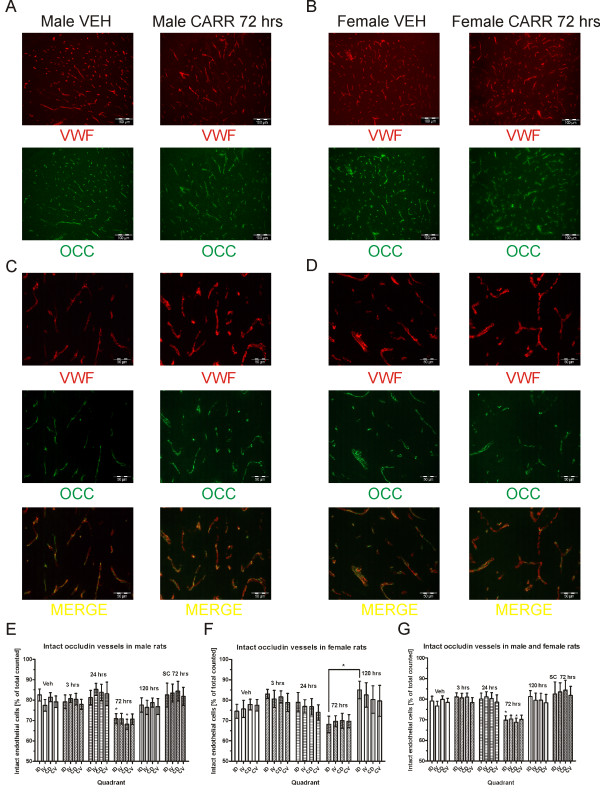
**Intraplantar carrageenan results in significant disrupted spinal occludin in dorsal horn.** Intraplantar carrageenan results in the appearance of disturbed occludin protein at 72 hours post-carrageenan in male (**A**) and female (**B**) rats. High magnification pictures show diffuse occludin staining and missing areas of overlap between occludin and VWF staining in both male (**C**) and female (**D**) rats. Blinded quantification reveals significant reduction of intact ipsilateral dorsal and contralateral dorsal occludin at 72 hrs post-carrageenan in male rats (**E**), but not in female rats (**F**). Pooled male and female groups show significant differences between vehicle and the 72 hour post-carrageenan treatment in dorsal horn (**G**). Subcutaneously administered carrageenan away from the hindpaw does not cause alterations in occludin protein. **Abbreviations:** subcutaneous (SC), others as in Figure 1 and 2.

We then looked at the effect of 3% carrageenan on the endothelial cell occludin protein at various timepoints in male and female rats. Here, significant occludin disturbance was noted at the 72 hour timepoint in both male (Figure 3A) and female (Figure 3B) rats. High magnification pictures show occludin disturbance manifesting as either diffuse/foggy and missing partially or fully as compared to endothelial cell staining (Figure 3C and Figure 3D). In order to quantify the occludin disturbance, a total of 11010 vessels were counted in the 6 male vehicle/carrageenan groups (mean of 1835 ± 231 per group) and 8489 vessels in the 5 female vehicle/carrageenan groups (mean of 1698 ± 267.5) in a randomized and blinded fashion. Two-way ANOVA of quantified vessels in male rats revealed a highly significant effect of group (F_5, 120_ = 28.35; P < 0.001), but not region or interaction. The 72 hour post-carrageenan timepoint was significantly different from vehicle treated group (P < 0.05) (Figure 3E). In order to confirm that the effect was due to peripheral hindpaw inflammation rather than a systemic inflammation, the effect of 3% carrageenan was also compared when injected subcutaneously in the dorsum. Here, the number of disturbed occludin vessels after systemically-administered carrageenan was not found to be significantly different from vehicle (P > 0.05). Comparing female rats, two-way ANOVA also revealed a highly significant effect of group (F_4, 84_ = 19.85; P < 0.001), but no effect of region or interaction (P > 0.05). However, there were no significant differences between vehicle and any carrageenan treatments (P > 0.05) (Figure 3F). A comparison between the 72 hour timepoint and the 120 hour timepoint revealed a significant difference for the ipsilateral dorsal region (P < 0.05) but no other comparisons, explaining the group effect detected by the ANOVA. While a trend for decreased intact occludin at 72 hours post-carrageenan similar to the male rats was noticeable in the female rats, the variability, in general, was greater particularly in the vehicle-treated animals. Therefore, we decided to combine the male and female animals and reanalyzed our data. Again, ANOVA showed a highly significant effect of group (F_5, 224_ = 20.72; P < 0.001). The post-hoc test revealed a significant difference only between the vehicle and the 72 hour post-carrageenan treatment in the ipsilateral and contralateral dorsal regions (P < 0.05) (Figure 3G). Hence, occludin protein on endothelial cells in lumbar dorsal horn is significantly disturbed only at the 72 hour timepoint after carrageenan, an effect that is likely to be due to the hindpaw inflammation and not a systemic inflammatory effect.

**Figure 4:**
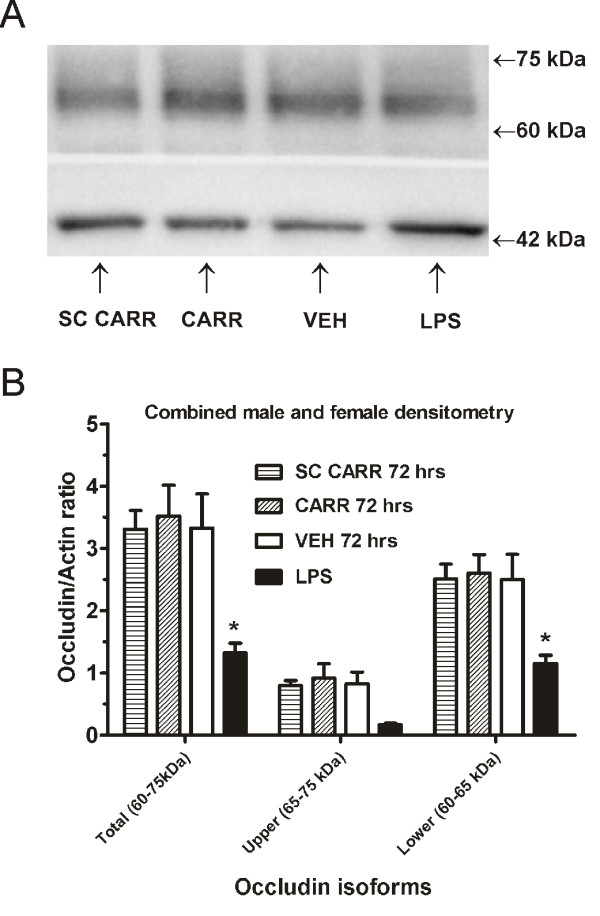
**Intraplantar carrageenan does not result in significant reduction in lumbar spinal occludin protein.** Intraplantar carrageenan does not result in the reduction of spinal occludin protein at 72 hours post-carrageenan in male and female rats as compared to vehicle, but this is detected after intrathecal LPS. (**A**). Densitometric quantification reveals significant decreases in the occludin/actin ratio after LPS, but not at 72 hours post-carrageenan as compared to vehicle (**B**). **Abbreviations:** female (FEM), others as in Figure 1, 2, and 3.

We also performed Western Blot staining for occludin protein from whole lumbar spinal cord. We used the experimental autoimmune encephalitis (EAE) model as a positive control since this has been previously shown to show altered occludin protein levels [6]. We confirmed a decrease in occludin in the 10 day EAE animals (data not shown) and also in the intrathecal LPS group (Figure 4A). However, we could not detect any significant differences in occludin protein levels in the 72 hour post-carrageenan groups. Quantification was performed by densitometry by pooling male and female groups in order to increase power and taking the ratio of occludin to its respective loading control (β-actin) after background subtraction in the following protein band regions: total occludin isoforms (~65-79 kDa), lower isoforms (~65-70 kDa), and higher isoforms (~70-79 kDa). This differentiation was performed due to the reports of occludin tendency to phosphorylate [6,25] and since the antibody used is reported to bind to the different molecular-weight isoforms. One-way ANOVA comparison of occludin/actin ratios revealed a significant effect of group for the total occludin ratios (F_3, 27_ = 3.34; P < 0.05) and lower isoforms (F_3, 27_ = 3.09; P < 0.05). Post-hoc tests revealed significant differences between intrathecal LPS and all other groups, but no differences between vehicle and carrageenan groups (Figure 4B). Taken together, our results suggest that there are morphological changes in occludin protein in lumbar spinal quadrants, but no detectable change in the protein level in the entire lumbar spinal cord.

### Intraplantar carrageenan induces IgG extravasation at 72 hours post-carrageenan

**Figure 5:**
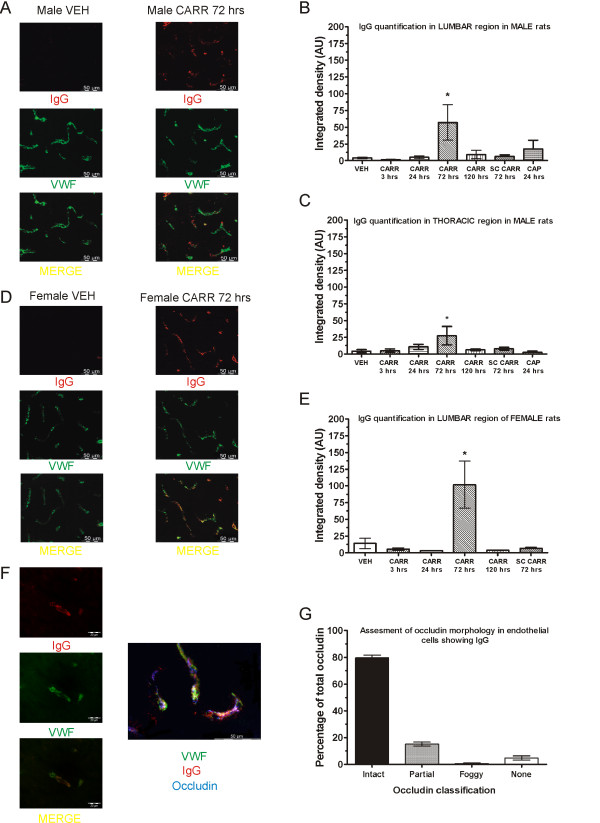
**Intraplantar carrageenan induces significant IgG extravasation in spinal cord.** Intraplantar carrageenan induces the appearance of IgG in endothelial cells and nervous tissue at 72 hours post-carrageenan in both male (**A**) and female (**D**) rats. Quantification reveals significantly increased fluorescence intensity in both male (**B**) and female (**E**) rats at this timepoint, but not with dorsally-injected subcutaneous carrageenan, or with capsaicin. Significant IgG is also detected in the thoracic region which correspondingly also peaks at 72 hours post-carrageenan (**C**). High magnification picture and triple staining of occludin, VWF, and IgG are shown (**F**). Endothelial cells with IgG staining show intact occludin protein within a normal range (**G**). **Abbreviations:** Immunoglobulin (IgG), others as in Figure 1, 2, and 3.

Since a significant occludin disturbance was found at 72 hours post-carrageenan, the effect of intraplantar carrageenan was next examined with another method suggestive of BSCB breakdown, IgG extravasation in spinal cord. By performing a long perfusion with phosphate buffered saline (PBS) to washout the blood, it was expected that any IgG in the blood vessels would be cleared. Double immunolabeling for IgG and WWF was then performed in lumbar and thoracic spinal sections. Interestingly, as with occludin disturbance, intraplantar carrageenan was found to induce significant extravasation and endothelial cell IgG accumulation only at the 72 hour timepoint in both male (Figure 5A) and female rats (Figure 5D). High magnification pictures show that the IgG appears to be mostly located close to the endothelial cell (Figure 5F), although sometimes it was located outside. In comparing fluorescence intensity values, one-way ANOVA revealed a significant group effect for both male (F_6, 31_ = 2.58; P < 0.05) and female animals (F_5, 28_ = 5.48; P < 0.05). Post-hoc tests showed significant differences between vehicle treatment and the 72 hour timepoint in both male (P < 0.05) (Figure 5B) and female animals (P < 0.05) (Figure 5E). We also did not detect significant IgG accumulation at the 24 hour capsaicin timepoint. In order to ascertain that hindpaw inflammation itself causes the IgG accumulation and not the dose used, carrageenan was also administered subcutaneously in the dorsum as in the previous section. These animals also did not show any significant IgG accumulations (P > 0.05).

As with the occludin study, IgG appeared to show effects that were not restricted to the ipsilateral dorsal horn. Hence, we also searched for IgG accumulation in the thoracic region and found that it was also present at the 72 hour post-carrageenan timepoint in several animals which also had lumbar IgG staining. One-way ANOVA revealed a non-significant trend group effect (F_6, 28_ = 2.3l; P > 0.05), although the post-hoc test revealed significantly increased fluorescence intensity at 72 hour post-carrageenan as compared to vehicle (P < 0.05) (Figure 5C). This suggested a spread of this effect beyond the lumbar spinal cord, but again not seen with systemically-administered carrageenan.

In the 72 hour post-carrageenan groups, IgG staining appeared to be mostly restricted to endothelial cells, as demonstrated by VWF staining. However, in some cases, it was clearly present outside the endothelial cells suggesting some extravasation does occur (Figure 5A and Figure 5B). We could not systematically detect IgG inside neurons as we saw in the EAE model (data not shown), suggesting that extravasation was mild. In order to search for a common mechanism for BSCB breakdown, we also performed triple staining with VWF, IgG, and occludin in the 72 hour treated carrageenan male and female rats (Figure 5G). By selecting vessels that showed IgG extravasation, we blindly assessed the status of occludin protein (as above) in 206 vessels from 3 male and 3 female rats. While we expected the number of disturbed occludin protein to be particularly high in these vessels, it was surprisingly within the range previously measured with double staining. This suggested to us that occludin disturbance and IgG accumulation are distinct mechanisms which occur independently.

### Histological assessment, Evans blue, and sodium fluorescein dye leakage

We then used other methods to assess the BSCB by searching for spinal cord structural changes by histological staining [26] and measuring the extravasation of intravenously administered small molecule fluorescent dyes such as Evans Blue and sodium fluorescein (NaFl) [19,27].

**Figure 6:**
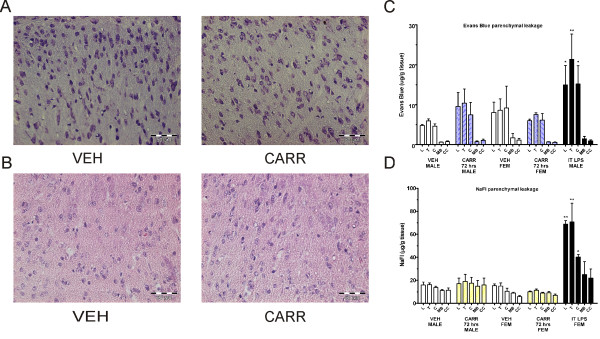
**No major histopathological changes, sodium fluorescein, or Evans Blue dye extravasation detected at 72 hours after intraplantar carrageenan.** H&E staining (**A**) and Nissl staining (**B**) do not reveal major lumbar spinal cord structural abnormalities at 72 hours post-carrageenan as compared to vehicle. There is no significant detection of extravasated Evans Blue (**C**) or NaFL (**D**) dye in spinal cord or brain in neither male nor female rats at 72 hours post-carrageenan as compared to vehicle treatment. Intrathecal LPS injected animals do show significant leakage of dye in lumbar, thoracic, and cervical regions. Abbreviations: lumbar (L), thoracic (T), cervical (C), midbrain (MB), cerebral cortex (CC), others as in Figure 1, 2, 3, and 4.

Simple Nissl staining (Figure 6a) and H&E staining (Figure 6b) were performed on paraformaldehyde (PFA)-perfused and fixed sections of both male and female vehicle-injected and 72 hour post-carrageenan rats. Multiple sections were blindly examined with the help of an experienced histologist. No major abnormalities were found between vehicle and carrageenan rats. There was occasional evidence of swollen perivascular space seen with Nissl staining. Although not systematically quantified, this was deemed to be a probable perfusion artifact rather than a change, as it was also seen occasionally on vehicle injected rats.

In separate rats, NaFl (molecular weight of 376 Daltons) or Evans Blue (molecular weight of 960 Daltons) dyes were administered intravenously and allowed to circulate for at least 30 minutes under inhalable anaesthesia. Leakage into the parenchyma of lumbar, thoracic, cervical, midbrain, and cerebral cortex was then quantified in vehicle-injected, 72 hour post-carrageenan injected male and female rats, and intrathecal LPS controls. A clear yellow staining for NaFl or blue staining for Evans Blue was visually detected in the spinal cord segments upon removal in LPS injected rats. For Evans Blue, two-way ANOVA revealed a significant effect of group (F_4, 70_ = 5.63; P < 0.05), and region (F_4, 70_ = 14.1; P < 0.05). Significant differences were detected by posthoc between LPS and vehicle groups in the lumbar (P < 0.05), thoracic (P < 0.001), and cervical (P < 0.05) regions, but not between vehicle and carrageenan treatment group. A similar pattern was seen in separate experiments using NaFl dye injection. Two-way ANOVA of fluorescence values revealed a highly significant effect of group (F_4, 65_ = 28.28; P < 0.001), region (F_4, 65_ = 7.57; P > 0.05) and interaction (F_16, 65_ = 2.38; P > 0.05). Significant differences detected by posthoc from the LPS and vehicle rats were seen in the lumbar and thoracic regions (P < 0.001), also significant in the cervical region (P < 0.05), but not in brain regions (P > 0.05). There were no significant differences at the 72 hour timepoints in neither the male nor the female carrageenan rats (Figure 5). Similar results had also been obtained with *in vivo* imaging using rhodamine-based dyes (data not shown). These results suggested to us that the above methods to assess the BSCB involve different mechanisms than tight junction protein or endogenous IgG assessment and emphasizes the importance for the use of multiple methods to assess the BSCB after transient pathology. In summary, changes in spinal occludin and presence of endothelial and/or parenchymal IgG can be detected transiently at 72 hours post-carrageenan, but not fluorescent dye extravasation or lumbar spinal cord structural changes which may all be necessary to conclude a “breakdown” in the BSCB.

## Discussion

Altered blood-CNS barriers can involve transient and specific changes with a complex functional effect on permeability of endogenous substances, immune cells, or drug treatments. Alterations in specific mechanisms such as changes in transporter proteins, tight junctions, and immune cell penetration can occur quickly in various conditions such as stress or xenobiotic exposure [28-30]. Transient changes are less studied than those that cause a clear “breakdown” of the BSCB in chronic diseases, those that can impact the BSCB for weeks and months such as spinal cord trauma, CNS inflammation, or peripheral nerve injuries. In the current study, we studied capsaicin neurogenic inflammation which induced mechanical hyperalgesia that peaked at about 2–3 hours and lasts for at least 24 hours, and carrageenan inflammation which induced mechanical and heat hyperalgesia that peaked at about 3–24 hours and lasts for at least 72 hours. Decreased intact occludin morphology was found in male rats and IgG extravasation in lumbar and thoracic spinal cord in both male and female rats. Interestingly, these effects were only detected at 72 hours post-carrageenan, a late timepoint, one which is beyond the peak hyperalgesia in this model and in which the inflammation is subsiding. As we did not find increased extravasation of exogenously administered small size fluorescent dye either Evans Blue or sodium fluorescein at ~30 minutes post-administration in these animals, this suggests that there is no generalized “breakdown” of the BSCB.

In the first experiments, we found that intact occludin on endothelial cells was disrupted after intraplantar carrageenan administration at 72 hours post-administration in male rats, but not after capsaicin administration or subcutaneous carrageenan. Various studies have shown that the presence of occludin protein determines tight junction permeability [31,32] and hence its disruption will influence the BSCB. Several chronic disease models show decreases in total spinal occludin protein such as EAE [33], diabetes [34], and nerve injury [19]. Intraplantar carrageenan has been shown to induce a biphasic effect on radioactive glucose permeability in the brain which parallels decreases in occludin from isolated microvessels with a peak at 1–6 hours post carrageenan and also at 48 hours post-carrageenan [35]. Interestingly, while we found decreased intact occludin morphology after carrageenan, we did not find a corresponding decrease in occludin protein and we also did not find evidence for a biphasic effect. It is possible that we were unable to detect changes in specific isoforms. Occludin has different phosphorylation sites and the highly phosphorylated occludin form has been suggested to be the functional form of the protein [36,37]. Peripheral carrageenan has been shown to induce changes in the relative amount of oligomeric, dimeric, and monomeric isoforms of occludin associated with brain endothelial cells [25]. However, it is also interesting to note that accumulating evidence suggests that there may be various differences between the BBB and BSCB such as, for example, differences in transporter and occludin expression [38] or regional differences in penetration of radioactive tracers and cytokines [39,40]. It would be interesting for future studies to simultaneously compare the timecourse of changes in both spinal and brain regions. Nevertheless, after EAE inflammation, dephosphorylation of spinal occludin has been shown to coincide with disruption in occludin morphology [6]. As we did detect decreased total occludin levels in the spinal inflammation positive controls, but not carrageenan, this may suggest that morphological changes may be more sensitive indicators of early BSCB disruption.

The timing of occludin morphology disturbance 72 hours after carrageenan was paralleled by the presence of IgG in endothelial cells and parenchyma. IgG is a large ~150 kDa molecule that under normal conditions does not cross the BSCB. Its presence in the cerebrospinal fluid has been labeled as a marker of disease clinically [41]. Various disease models such as apolipoprotein-E knockout mice [42], rat spinal cord injury [43], and partial sciatic nerve ligation [19] show IgG accumulation in the spinal cord. In the superoxide dismutase-1 mutant rat model of amyotrophic lateral sclerosis, IgG is also reported in the lumbar spinal cord and cortex, although interestingly at the pre-symptomatic stage prior to changes in occludin and increased penetration of Evans Blue dye [44]. We did detect IgG in the nervous tissue, although a large part of the IgG appeared to compartmentalize or stay within the endothelial cells in the carrageenan treated animals. Given that our vehicle-treated animals did not show IgG and the effect occurred only at 72 hours post-carrageenan, we do not believe that this is an artifact of perfusion or due to vascular abnormalities. It would be interesting to speculate that this IgG accumulation in endothelial cells may signal an early change of the BSCB which does not progress to a more generalized “breakdown”. Interestingly, IgG was further detected in the thoracic spinal cord indicating a widespread effect. Evans Blue extravasation after electrical C-fiber stimulation of the sciatic nerve or after peripheral nerve injury is also reported to occur in the thoracic spinal cord [20] and our changes in occludin protein were not restricted to the ipsilateral dorsal horn. It is also known that spread of BSCB changes can occur in a delayed manner after spinal cord injury [45] and that hyperalgesia can spread beyond the injury site in various animal models. Although we did not detect significant contralateral nociception in our animal model, several potential mechanisms for spreading and secondary hyperalgesia could also be involved here to explain why changes would not be restricted to the ipsilateral lumbar dorsal horn. These include activation of the astrocytic network, disinhibition, infiltration of immune cells, volume transmission in the CSF, heterosynaptic long-term potentiation, involvement of commissural interneurons, and of descending facilitation [46]. It can also be speculated that there may be a delayed release of a humoral mediator. However, it should be emphasized that carrageenan administered subcutaneously did not induce any IgG accumulation at 72 hours post-administration. Hence, it is likely that the peripheral ongoing nature of the inflammation in the hindpaw tissue is responsible for the late-occurring IgG extravasation which is clearly different from a systemic or CNS inflammation. Nevertheless, it is intriguing to consider how other peripherally administered agents beyond carrageenan may also have subtle and spreading CNS effects beyond the usual endpoints measured related to hindpaw nociception and the lumbar dorsal horn.

Testing the permeability of the BSCB after injection of the small molecular weight NaFl did not reveal an increased permeability at the 72 hour post-carrageenan timepoint in neither male nor female rats. Most studies on barriers have traditionally used Evans Blue which has a higher molecular weight than NaFl. Leakage has been shown to occur as early as 3 days after nerve injury [19] or 24 hours after capsaicin or sciatic nerve stimulation [20]. Interestingly, carrageenan was reported to induce spinal Evans Blue leakage in one study [17], although neither brain nor spinal cord leakage in another [18]. *In vitro*, endothelial cells treated with adrenomedullin show increased permeability to NaFl, but not Evans Blue [47]. Also *in vivo* after nerve injury, NaFl shows greater differences in leakage as compared to Evans Blue [19]. Hence, sensitivity is likely higher using the NaFl dye. However, it is also known that NaFl binds to transport proteins such as organic anion transporter [48,49]. In streptozotocin-treated diabetic rats, there is increased BBB permeability as evidenced by increased penetration of radioactive sucrose and decreased tight junction protein [50], but actually decreased permeability to fluorescein explained by upregulation of transporters in this model [51]. Increased morphine penetration is also reported in peripheral inflammation which does not coincide with increased Evan Blue dye penetration [18]. It is interesting to speculate whether the reported increased systemic analgesic sensitivity after inflammation for opioids [52] or other drugs [53] can be due only to changes in the BSCB as detected in our study.

In addition, we performed hematoxylin/eosin and Nissl staining in lumbar spinal cord in 72 hour animals. Previous reports have indicated structural alterations in the spinal cord after nerve injury [26] and in the brain with acute stimuli such as hyperthermia and methamphetamine administration [54,55]. We did not detect such extensive changes as in the literature in the 72 hour post-carrageenan group, and although we did find occasional instances of perivascular edema, this was not systematically quantified and electron microscopy was not used in our study. Future comprehensive studies to quantify the presence of minor structural abnormalities may be interesting to establish the sensitivity of this method for detecting more subtle changes in the BSCB.

We hypothesized that we could detect sex differences in the BSCB. As within the pain literature [56,57], there are reports of sex differences in the BBB which prompted us to examine this topic for the first time in this context. Effects of sodium selenite and antioxidants are reportedly greater in males than in females which have greater alterations of BBB permeability in some models of epilepsy [58,59]. However, other studies show greater permeability in female mice to selenium as compared to male mice during systemic inflammation [60]. Radio frequency radiation also appears to show BBB disruption in male rats but not female rats [61]. Interestingly, multiple studies that have found changes in the BBB after peripheral carrageenan were performed only in female rats [62,63]. In our study, we detected a significant difference in male rats in the occludin study, however both for male and females in the IgG study. This appeared to us to be due to greater baseline variability in females. It is possible that particular estrous cycle stages may influence occludin morphology. The effect of lifetime estrogen changes and exogenous estrogen administration has been speculated to influence BBB penetration, loss of occludin protein, and appearance of IgG [23,64]. It would be interesting for future studies to systematically examine whether particular estrous cycle phases may alter susceptibility to changes in BSCB.

The timecourse of the changes seen in occludin morphology and IgG extravasation in our study does not provide a direct correlation between the initiation or peak nociception and changes in the BSCB, since carrageenan hyperalgesia peaks within a few hours. This is intriguing given that direct electrical stimulation of nociceptive C-fibres of the sciatic nerve causes a widespread opening of the BSCB prevented by locally applied lidocaine [20] or the prevention of BBB permeability and tight junction proteins alteration by bupivacaine nerve block [63]). Animal models of peripheral inflammatory pain show specific changes in the BBB which can occur shortly after the stimulus when nociception is present. Injection of 5% formalin, 3% carrageenan, or CFA induce changes in brain tight junction proteins and increased brain permeability to radioactive sucrose at 1 hour, 3 hours, and 48 hours [35,65,66]. Whereas anti-inflammatory treatments such as rapamycin or diclofenac reduce blood-CNS barrier breakdown together with hyperalgesia [62,67], no studies establish BSCB alterations as necessary and sufficient for nociception. Changes in the BSCB may also be secondary effects nonspecific to nociception and as other indicators of central inflammation may be dissociable from measurable nociceptive behaviors [68,69]. In the current study, the doses of capsaicin and carrageenan used were on the high range as compared to the literature making it unlikely that lower or more commonly used doses of capsaicin or carrageenan would have elicited more extensive changes on the BSCB. If due to the nociceptive barrage, early effects should be expected in the first synapse of spinal cord, but we could not detect this with capsaicin or early after carrageenan. It can also be speculated that changes detected at 3 days post-carrageenan are not related to peak nociception in peripheral inflammatory pain, but perhaps to compensatory “healing” mechanisms. Our study used multiple methods to assess whether there is “breakdown” of the barrier and suggests that each method may detect particular mechanisms involved in changes in the BSCB.

## Conclusion

In summary, we have found evidence of changes in spinal occludin protein and appearance of IgG around endothelial cells occurring with a delayed onset after peripheral inflammation and nociception. These changes are transient and are not restricted to spinal cord areas of nociceptive input. We conclude that peripheral inflammation induces transient changes in the spinal cord indicative of an altered BSCB but these do not appear linked directly to nociceptor activation.

## Methods

### Animals

Male and female Sprague–Dawley rats (175–250 grams) were used in all experiments and obtained from the Medical University of Vienna local breeding facility (Himberg, Austria), except in EAE complex inflammation where Lewis rats were used (obtained from Charles River, Germany). Rats were housed 4–6 per cage in a unisex manner under 12/12 light dark cycle at temperatures between 20-25°C and humidity between 40-60%. Food and water was provided ad libitum. Animals were handled at least twice prior to use and care was taken to minimize stress during all experimental procedures. All experimental procedures were approved by the ethics committee of the Medical University of Vienna (MUW) and the Austrian Ministry for Science and Research (BMWF), and conform to the animal experimentation standards of the International Association for the Study of Pain (IASP).

### Animal models

Capsaicin (1% sonicated in mild heat in 80% 0.9% NaCl, 10% pure ethanol, 10% Tween-80; Sigma) was injected into the hindpaw at a volume of 25 μl under inhalable anaesthesia with oxygen, nitrous oxide (N_2_O) and isoflurane. λ-carrageenan (3% mixed vigorously in NaCl) was injected into the hindpaw at a volume of 100 μl also under gas anaesthesia. In some groups, carrageenan was administered in the nape of neck subcutaneously at the same volume and dose. As positive controls for spinal inflammation, LPS was injected intrathecally as per [70] with a first injection of 2 μg/30 μl followed by a second injection of 20 μg/30 μl 24 hours later. The animal was sacrificed 3 hours after the second injection. For the EAE positive control, male and female Lewis rats were used as previously described [71] and sacrificed 7–10 days after induction of complex inflammation.

### Behavioral assessment

In the first group of experiments, male and female rats (n = 6 per group) were injected intraplantar with 1% capsaicin or vehicle, and nociception was measured at 1, 2, 3, 4, and 24 hours post injection. Mechanical thresholds were measured using a set of calibrated Von Frey filaments (Stoelting Europe, Dublin, Ireland) between 0.4 grams and 15 grams with animals standing on a wire mesh inside clear Plexiglas boxes. The caudal region behind the ipsilateral footpad was stimulated three times using a three-second stimulation. A positive response was counted when a clear hindpaw withdrawal occurred, not due to movement of the animal. Ascending hairs were presented in a systematic manner starting with the 2 gram hair. After the first response was obtained, four additional hairs were presented using the up-down method based on [72]. The 50% paw withdrawal threshold was then interpolated using the formula: 50% g threshold = (10^[xf +k*δ*]^)/10,000, where *x*_f_ = value (in log units) of the final von Frey hair used; *k* = tabular value (see [72] for pattern of positive/negative responses; and *δ* = mean difference (in log units) between stimuli in each pattern used and stimulation in adjacent areas in this same region. The contralateral paw was always tested first followed by the ipsilateral paw.

In the second group of experiments, male and female rats (n = 6 per group) were injected intraplantar with 3% carrageenan or vehicle, and nociception was measured at 3, 24, 48, and 72 hours post-injection. Mechanical thresholds were measured using a calibrated forceps (Bioseb, Vitrolles, France). Habituated animals were briefly and lightly restrained in a soft towel inhibiting visual stimulation. The hindpaw was progressively squeezed at a rate of approximately 100 g per second. The mechanical threshold was electronically obtained when the animal clearly withdraw its hindpaw and/or vocalized. Three readings per hindpaw for the baseline and two readings per hindpaw for the timepoints post-injection were measured with a 10-minute inter-testing interval. Thermal latencies were measured using the plantar Hargreaves Apparatus (Stoelting Europe, Dublin, Ireland). The animals were placed on a heated glass floor (30°C) and the time latency to hindpaw withdrawal (not due to movement) was measured with application of the light source on the region caudal to the foot pads. The radiant heat intensity was set to an arbitrary value that elicited a baseline withdrawal latency of about 10–12 seconds at the beginning of the experiment. Three baseline readings were performed and averaged for analysis. Thereafter, two readings per hindpaw were averaged for the timepoints post-injection with a 10 minute inter-testing interval. The contralateral paw was always tested first followed by the ipsilateral paw.

### Immunohistochemistry

#### Occludin and VWF staining and quantification

For capsaicin experiments, male (n = 4-6 per group) and female (n = 4-5) animals were sacrificed at 10 minutes, 3 hours, 24 hours and 72 hours (only in males) after 1% capsaicin or vehicle injection. For λ-carrageenan experiments, male (n = 5-8 per group) and female (n = 5-6 per group) animals were sacrificed at 3 hours, 24 hours, 72 hours, and 120 hours (only in males) after λ-carrageenan or vehicle injection. To collect the spinal cord, animals were deeply anaesthetized using ether and decapitated. The spinal cord was removed immediately using pressure expulsion by injecting cold PBS in the spinal column at the sacral level using a 18-gauge needle. Thereafter, the lumbar region was carefully dissected, snap-frozen in isopentane, and stored at −80°C. 14-μm-thick transverse slices of the L5 lumbar region were sectioned at −20°C and placed on slides. After drying, slides were fixed in 100% ethanol for 30 minutes at 4°C and then acetone for 3 minutes at −20°C. Thereafter, slides were washed once with PBS and blocked with 5% normal donkey serum (NDS) at room temperature for 30 minutes. Sections were then incubated with the primary antisera mouse anti-occludin (1:1000 dilution in 5% NDS; Invitrogen) and rabbit anti-VWF (1:4000 dilution in 5% NDS; Sigma-Aldrich) for 1 hour at room temperature. Afterwards, the sections were washed with PBS and incubated with the secondary antibodies goat anti-mouse Cy2 (1:200 in PBS; Jackson Immunoresearch) and donkey anti-rabbit Cy3 (1:400 in PBS; Jackson Immunoresearch) for 1 hour at room temperature. After final washing with PBS, slides were coverslipped with Aquatex® (Merck Millipore, Darmstadt, Germany).

To investigate occludin expression, the sections were analyzed using a fluorescence microscope (Olympus BX51) and pictures were acquired using a colour fluorescence camera (Olympus DP50). Discrete occludin was counted in every spinal cord quadrant (ipsilateral ventral, ipsilateral dorsal, contralateral ventral, contralateral dorsal) in comparison to the VWF staining, and the expression within each vessel was manually classified as “intact” (clear lines of occludin), “partial” (less than 80% occludin per vessel), “foggy” (diffuse distribution of occludin) and “lacking” (no occludin) by an experimenter blinded to the sample identity. Between ~60 and 100 vessels per quadrant were randomly counted with this method.

#### IgG staining

Male (n = 4-5 per group) and female (n = 4-6 per group) animals were used that had undergone the following treatments: vehicle, 3 hours post-carrageenan, 24 hours post-carrageenan, 72 hours post-carrageenan, 120 hours post-carrageenan, subcutaneous carrageenan (72 hours post), and 24 hours post-capsaicin. Rats were deeply anaesthetized using ether and perfused intracardially at a rate of ~10 ml/min using a peristaltic pump with 150 ml oxygenated PBS which was warmed to 37°C. The spinal cord was then immediately removed by pressure expulsion with cold saline and snap-frozen in isopentane and stored at −80°C. 14-μm-thick transverse slices of the L5 lumbar region and T3 thoracic regions were sectioned using a cryotome at −20°C. After drying for 1 hour, the slides were fixed in 100% ethanol for 1 hour at 4°C and for 3 minutes in acetone at −20°C. After washing with PBS, they were blocked with 5% NDS in PBS with 0.1% TritonX-100 for 30 minutes. Sections were then incubated in rabbit anti-VWF (1:4000) and donkey anti-Rat IgG Cy3 (1:600; Chemicon) over night at 4°C. The sections were then washed with PBS and incubated for 1 hour at room temperature with the secondary antibodies: donkey anti-rabbit Cy2 (1:200 in PBS) and again donkey anti-rat IgG Cy3 (1:600 in PBS). Sections were washed again with PBS and the slides were coverslipped with mounting medium.

To analyze the IgG staining intensity as a marker for the BSCB integrity, we used a semi-quantitative method. Pictures of the ipsilateral dorsal region of the slides were taken at 400X magnification with a fluorescence microscope and colour camera (Olympus BX51 and DP50) using a consistent exposure time. Three non-consecutive sections from each of the different groups were blocked and randomized per experimental session and the experimenter taking the photographs was blind to the experimental treatment. For analysis, a consistent colour threshold was set to subtract the background and the pictures were binarized using CellD 3.3 (Olympus Soft Imaging Solutions). The integrated density (area X mean grey value) of the whole images was measured using ImageJ software (National Institute of Health) and averaged per group.

#### Triple staining-occludin, IgG, and VWF

A similar procedure as the IgG/VWF was used. The antibodies used in this case were primary guinea pig anti-Occludin (1:10; Hycult Biotech), rabbit anti-VWF (1:4000) and donkey anti-Rat IgG Cy3 (1:600) overnight at 4°C. All antibodies were diluted with 0.1% PBST + 5% NDS. To investigate a direct correlation between IgG extravasation and occludin distribution, lumbar sections from male and female 72 hours post-carrageenan groups (n = 6) were used and occludin protein in those endothelial cells co-staining with IgG from the ipsilateral dorsal regions was classified as above into intact, partial, foggy, or no staining. 15–40 vessels per animal were analyzed. Representative pictures of the triple staining were made with the monochrome fluorescence camera (Olympus XM10).

### Western blot for occludin

Male and female animals (n = 4-8 per group) treated with either vehicle, 72 hours post-carrageenan, or 72 hours after subcutaneous carrageenan were used in these experiments. Male intrathecally injected LPS (n = 4 per group), naive and EAE animals were used as control groups for the assay. Rats were decapitated and the spinal cord was removed by cold PBS pressure expulsion. The lumbar spinal cord (L4-L6) was snap frozen at −80°C in isopentane and stored at the same temperature until further processing. Tissue samples were homogenized in Lämmli-buffer including protease inhibitors (Complete Mini, EDTA-free, Roche), heated to 70°C for 10 minutes, vortexed, heated to 95°C for 5 minutes and centrifuged for 10 minutes at 4°C and 14.000 *g.* Supernatants were then collected and equal amounts of protein were size fractioned by SDS-PAGE (8% gel) using the Mini-Protean 3 Cell- system (Bio-Rad) and transferred onto a nitrocellulose membrane (Protran, Whatman). The membrane was placed in blocking buffer, consisting of dry milk powder and Tween-80 (Sigma) in phosphate buffered saline for 1 hour and then incubated over night at 4°C with primary polyclonal antibody to Occludin (rabbit anti-occludin 1:700, Invitrogen). The expression of ß-Actin (monoclonal anti-ß-actin 1:700, Sigma) was used as an internal loading control. All antibodies were diluted with blocking buffer. The blots were washed three times for 15 minutes each and then incubated with peroxidase-conjugated anti-rabbit IgG and peroxidase-conjugated anti-mouse IgG, respectively, for 2 hours at room temperature. Secondary antibodies were purchased from Jackson Immuno Research. After washing the blots three times for 15 minutes each, the bands were visualized by the Immobilon Western Chemiluminescent HRP Substrate (Millipore) and detected by the Fluor-S MultiImager (Bio-Rad). Densitometric quantification of the bands was performed with the Quantity One software (Bio-Rad). Background was subtracted for each lane, and ratio between β-actin band density and pre-determined areas of low and high molecular occludin band density was determined by a blinded experimenter.

### Histological staining

Rats were deeply anaesthetized using ether and perfused intracardially first with 25 ml saline followed by 175 ml 4% PFA at a rate of ~10 ml/min using a peristaltic pump. The following groups (n = 3-4 per group) were collected: vehicle male, vehicle female, 72 hours post-carrageenan male, 72 hours post-carrageenan female, intrathecal LPS. The entire spinal cord column was dissected and post-fixed in 4% PFA overnight. The spinal cord was then removed by laminectomy and placed in 20% sucrose overnight, and 30% sucrose for 2 additional days, prior to being snap-frozen in isopentane and stored in −80°C until further processing. Frozen 8–10 μm sections from the lumbar spinal cord were then cut with a cryostat. Hematoxylin/Eosin and Nissl stainings were performed as per a standardized protocol on multiple sections per animal. Slides were coverslipped using Eukitt® (Sigma-Aldrich, Vienna, Austria).

### Evans blue dye and sodium fluorescein penetration assay

Male and female animals (n = 3-6 per group) from the following groups were used in this assay: vehicle, intraplantar carrageenan 72 hours post administration, subcutaneous carrageenan 72 hours post administration, and intrathecal LPS. Sodium fluorescein (10% in 2 ml/kg) (Sigma) or Evans Blue dye (3% in 4 ml/kg) (Sigma) was infused into a cannulated femoral vein and allowed to circulate for 30–40 minutes while the animal was kept under isoflurane/N_2_O anaesthesia. Animals were then immediately intracardially perfused with ~150 ml saline at a rate of ~10 ml/min using a peristaltic pump. Spinal lumbar, spinal thoracic, spinal cervical, midbrain, and cerebral cortex tissues were collected by cold saline pressure expulsion and dissection.

For NaFl measurements, tissues were weighed, homogenized, extracted in 60% trichloroacetic acid, and centrifuged at 18,000 G for 20 minutes. A standard curve with NaFl (in 60% trichloroacetic acid) was generated and fluorescence intensity was measured in supernatants (in duplicate) using a spectrophotometer at an excitation wavelength of 420 nm and emission wavelength of 525 nm. For Evans Blue dye measurements, tissues were weighed and extracted in formamide for 72 hours at 65^°^C. Tissues were then centrifuged at 18,000G for 20 minutes. A standard curve with Evans Blue dye (in formamide) was generated and fluorescence intensity was measured in supernatants (in duplicate) using a spectrophotometer at a wavelength of 620 nm. All measurements were converted to dye concentration per tissue weight.

## Competing interests

The authors declare that they have no competing interests.

## Author’s contributions

DNX carried out behavioral tests, tissue processing/immunostaining, data analysis, designed and coordinated experiments, and wrote the manuscript. IP carried out tissue processing/immunostaining and data analysis. GW carried out immunostaining and western blot, data analysis, and provided general technical support. JS conceptualized and directed the project. All authors read and approved the final manuscript.
